# Anti-proliferation effects of Sirolimus sustained delivery film in rabbit glaucoma filtration surgery

**Published:** 2011-09-27

**Authors:** Zhi-chao Yan, Yu-jing Bai, Zhen Tian, Hai-yan Hu, Xiu-hua You, Jian-xian Lin, Shao-rui Liu, Ye-hong Zhuo, Rong-jiang Luo

**Affiliations:** 1 Department of Ophthalmology, The First Affiliated Hospital, Sun Yat-Sen University, Guangzhou, Guangdong, People’s Republic of China; 2 Key Laboratory of Vision Loss and Restoration, Ministry of Education, Department of Ophthalmology, Peking University People’s Hospital, Beijing, People’s Republic of China; 3 State Key Laboratory of Ophthalmology, Zhongshan Ophthalmic Center, Sun Yat-sen University, Guangzhou, Guangdong, People’s Republic of China; 4 Research and Development Center of Pharmaceutics, School of Pharmaceutical Science, Sun Yat-sen University, Guangzhou, Guangdong, People’s Republic of China

## Abstract

**Purpose:**

To investigate the efficacy, safety, and mechanisms of Sirolimus sustained delivery film on prevention of scar formation in a rabbit model of glaucoma filtration surgery.

**Methods:**

Sixty-four New Zealand white rabbits who underwent trabeculectomy in the right eye were randomly allocated to one of the four treatment regimens: Sirolimus sustained delivery film treatment group (Group A), or drug-free film treatment group (Group B), or 30 ng/ml Sirolimus-soaked sponge treatment group (Group C), or no adjunctive treatment group (Group D), and each group consists of 16 rabbits. Intraocular pressure (IOP), morphologic changes of bleb, anterior chamber flare, and corneal endothelial cell count and complications were evaluated over a 28-day period follow-up time. Aqueous humor samples were gathered from Group A, and the concentration of Sirolimus was measured regularly post-operation. Rabbits were sacrificed on the 7th, 14th, and 28th day post-operation separately, and the fibroblast hypertrophy, infiltration of inflammatory, and proliferation of new collagen fiber formation in each group were evaluated with HE and Masson staining. Proliferative cell nuclear antigen (PCNA) and fibroblast apoptosis were evaluated by immunohistochemistry and terminal deoxynucleotidyl transferasemediated dUTP nick end labeling (TUNEL) assay at the 28th day post-operation.

**Results:**

Both Sirolimus sustained delivery film (Group A) and Sirolimus alone (Group C) were well tolerated in this model, and significantly prolonged bleb survival compared with no drug treatment group (Group B and D; p<0.001). Group A had the longest bleb survival time in comparison with other groups (p<0.001). There were significant differences in IOP readings between Group A and other groups at the last follow-up (p<0.05). The concentration of Group A maintained stable for over 2 weeks, drops from (10.56 ±0.05) ng/ml at day 3 to (7.74 ±0.05) ng/ml at day 14. The number of corneal endothelial cells of Group A was not statistically significant between pre and post-operation. Histologic examination demonstrated that eyes treated with Sirolimus, especially the Sirolimus sustained delivery film, showed an obvious reduction in subconjunctival fibroblast scar tissue formation compared with no drug treatment groups, and had minimal evidence of inflammatory cell infiltration and new collagen deposition in the subconjunctiva. Immunohistochemistry assay showed that PCNA-expression was lower in the Group A (16.25±3.24%) compared to other groups (p<0.01). TUNEL assay showed a significant increase in the number of apoptotic fibroblasts around the surgical area in Group A and Group C (9.75±1.71% and 8.50±1.92%) compared to the Group B and D (p<0.01).

**Conclusions:**

Sirolimus drug sustained delivery film can inhibit inflammatory cell activity, impede fibroblast proliferation activity, and induce fibroblast apoptosis in the filtration surgery sites in rabbit. The results indicate a safe and effective treatment strategy in anti-scaring treatment in glaucoma surgery.

## Introduction

Filtration surgery is currently one of the most effective methods to treat glaucoma. The most prevalent reason of failures in filtration surgery is post-operation scarring of the filtration path [1]. Due to overhealing of subconjuctiva at the bleb and sclerostomy sites, the failure rate of this surgery can reach 15%–30% [1,2]. Despite numerous anti-metabolites medications (e.g., Mitomycin C [MMC] and 5-Fluorouracil [5-FU]) greatly increased the success rate of glaucoma surgeries. However, these drugs may lead to increased incidences of complications, such as persistent postoperative hypotony, corneal problems, filtering bleb leakage, etc [3-6]. Therefore, developing a low toxicity, safe, effective and long lasting medication for anti-proliferation after filtration surgery has long been an important area of research for glaucoma.

Sirolimus, also known as Rapamycin (RAPA), is a natural product isolated from *Streptomyces hygroscopicus* [7]. It is a macrolide currently used as an immunomodulatory medication, an anti-tumor agent, or an anti-viral agent, featuring low toxicity and high efficiency [7-10]. Researches have indicated that Siromilus has a strong inhibition effect on the fibroblasts from the Tenon’s capsule through inhibit platelet-derived growth factor-induced fibroblast proliferation in vitro without any apparent cytotoxicity [11]. Thus it is possible for it to become an ideal anti-proliferation medication for glaucoma filtration surgery.

Until recently, the anti-metabolite medication was used transiently on the surgery site with a sponge soaked with special drug (MMC or 5-FU, etc) [3,4]. The application time and the concentration of these drugs had no identical criteria. Thus, the sustained drug delivery system (DDS) with constant effective concentration would be a useful way to get better clinical outcomes.

The purpose of this study is to investigate the effects of a Sirolimus sustained delivery film on the conjunctiva and scleral wound healing process after filtration surgery in rabbit eyes. We found this delivery system may be a safe and effective treatment to inhibit inflammatory cell activity and fibroblast activity in surgery sites, and can significantly improve outcome of filtration surgery.

## Methods

### Creation of the Siromilus sustained-delivery film

The drug delivery film was developed by the School of Pharmaceutical Science, Sun Yat-sen University (Guangzhou, Guangdong Province, P.R. China), and its production process is as follows: (1) Accurate weigh PEG4000 36 mg in 5 ml centrifuge tube, add 1.5 ml dichloromethane, vortex to dissolve. (2) Weigh polymer polyactioglyconic acid (PLGA, Durect Corporation, Birmingham, UK; inherent viscosity 0.55–0.75 dl/g, degeneration time: 8–12 weeks) 42 mg, PLGA (inherent viscosity 0.17 dl/g, degeneration time: 2–4 weeks) 15.6 mg, Siromilus (Hangzhou sino-us east China pharmaceutical Co., LTD, Hangzhou, China) 14 mg in 2 ml centrifuge tube, add 600 μl of the solution from (1), vortex 10 min to dissolve, set aside for 5 min. After the Siromilus was dissolved, the solution was dropped onto a slide in a ventilated hood until the solvent evaporates, and then the film was collected. Before application in the surgery, the film underwent ethylene oxide fumigation for 24 h in the dark.

### Animals and anesthesia

All animal experiments were conducted according to the guidelines of the ARVO Statement for the Use of Animals in Ophthalmic and approved by the Ethics Committee of Zhongshan Ophthalmic Center, Sun Yat-sen University.

New Zealand white female rabbits (weight, 2–2.8 kg; age, 10–12 weeks) were purchased from Guangdong Medical Laboratory Animal Center. Rabbits were anesthetized with an intramuscular injection of ketamine hydrochloride (50 mg/kg) and chlorpromazine hydrochloride (10 mg/kg) before surgeries and examinations. The right eye (OD) of each animal was used as experimental eye, and the contralateral left eye (OS) was considered as control. Sixty-four healthy rabbits were randomly divided into 4 groups (16 per group), and described as follow. (1) Group A: Sirolimus sustained delivery film implanted under conjuctiva; (2) Group B: Drug-free film implanted; (3) Group C: Sponge with a concentration of 30 ng/ml Sirolimus applied to the surgery site for 3 min and then wash out with Balance Saline Solution (BSS); (4) Group D: Trabeculectomy without adjunctive treatment. All investigations involving rabbits, including IOP measurements, slit lamp examination, corneal endothelial cell count, and determination of histologic features, were conducted in a blind manner by two experienced researchers.

### Filtration surgery protocol

**Figure 1:**
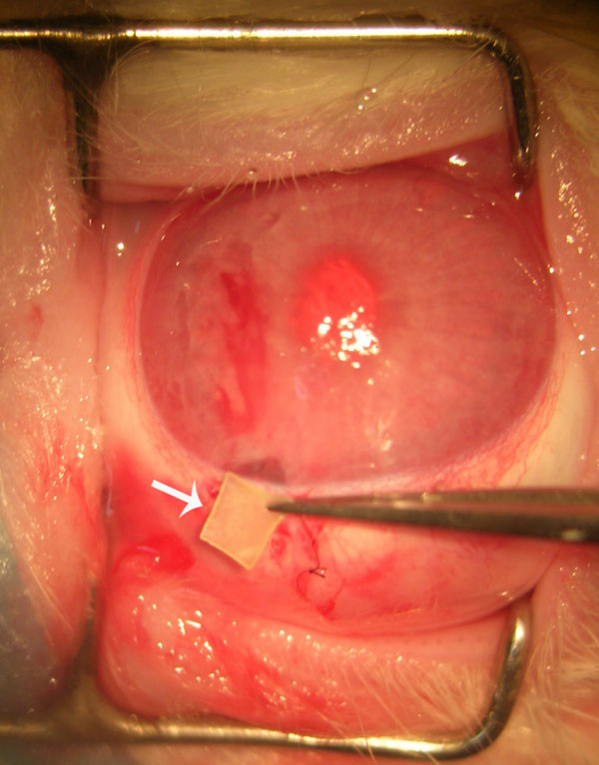
Sustained delivery film application during filtering surgery. A drug delivery film in white sheet form, approximately 2.5 mm×2.5 mm in size (arrow), placed in the surgical area between the conjunctiva flap and scleral flap.

After topical anesthesia with 0.5% tetracaine eye drops, a limbus-based flap of the conjunctiva and the Tenon’s capsule was made at a distance of 5 mm from the limbus in the superior quadrant of the right eye. After that, a half-thickness, rectangular, 4×3 mm scleral flap was created. A 2×1.5 mm scleroectomy was followed by peripheral iridoectomy. The scleral flap was closed with 10–0 nylon sutures. The conjunctiva was closed with a continuous locking suture with 8–0 Vicryl thread (Johnson & Johnson, Ethicon, NJ). Before suturing the conjunctiva flap, the Sirolimus sustained delivery film and blank film with the size of 2.5×2.5 mm were sutured onto the scleral which above the flap with 10–0 nylon thread at two cross corners in Group A and Group B, respectively (Figure 1). In Group C, cotton pad of size 4×3 mm with Siromilus concentrate of 30 ng/ml was applied between the scleral flap and conjunctiva flap for 3 min, and then rinsed with 200 ml BSS. In Group D, no adjunctive treatment was applied, and only treated with trabeculectomy.

After the surgery, treatment of TobraDex eyeointment was applied to the conjunctival sac. Post-operative treatment included tobramycin eyedrops (4 times daily), and tobramycin eyeointment (once per day) for a week. All operations and treatments were implemented by experienced ophthalmologists (R.J.L. and Y.H.Z.).

### Preoperative and postoperative examinations

#### Intraocular pressure (IOP)

Tono-Pen AVIA applanation tonometer was used to measure IOP during pre-op, post-op days 1, 3, 5, 7, 10, 14, 21, and 28 according to the manufacture procedure. Briefly, 0.5% tetracaine eye drops were applied on the tested eyes 2–3 times before measurements. After the rabbit’s head is stabilized, the tip of the measuring head was gently touch perpendicular onto the central corneal zone until results were shown, then were subsequently recorded (with ≥95% confidence interval). Each eye was measured 3 times, and the average value was taken.

#### Slit lamp examination

A slit lamp microscope was used to observe the eyes during post-op days 1, 3, 5, 7, 10, 14, 21, and 28. Eyes were observed for conjunctiva congestion, filtering bleb morphology and sustained time, anterior chamber flare and hyphema, cataract, and other such complications. Diffuse/elevated and cystic bleb was defined as functional bleb, while flat bleb was defined as failure bleb.

#### Corneal endothelial cell count

A TOPCON corneal endothelial cell count instrument (Topcon SP2000; Topcon, Tokyo, Japan) was used to determine endothelial cell count during pre-op and day 28 post-op according to the manufactures instruction. Briefly, the head of the rabbits were fixed in the chinrest of the machine. A confined central area within which all cells were marked was indicated. The integrated software of the microscope calculates the quantity of cells. At least 3 repeated counts were performed in each eye.

### Measurement of drug concentration in aqueous humor

At day 3, 7, 14, and 28 after filtration surgery, four rabbits in Group A were used for the measurement of the drug concentration under general anesthetic. Briefly, a 1 ml syringe was used under a ZEISS surgical microscope to biopsy 0.2 ml of aqueous humor, and HPLC-MS was used to determine the drug concentration in the aqueous humor.

### Histologic examination

Enucleation was performed on four randomly selected rabbits on days 7, 14, and 28 after surgery in each group. After enucleation, the eyes were immersed in 4% paraformaldehyde for 24–28 h at 4 °C. The tissue with the largest circumference in the filtering bleb area of the eye was resected and collected. After paraffin embedding, slide samples were collected, hematoxylin-eosin (HE) and Masson trichrome staining were performed. HE staining was used to observe conjunctiva epithelial integrity of the filtering bleb area, subconjunctival space, fibroblast hypertrophy, the degree of infiltration of inflammatory cells in the filtering bleb area, retinal involvement, and related changes [12-14]. Masson trichrome staining was used to further determine the proliferation of new collagen fibers in the bleb region [15,16].

### Immunohistochemistry and TUNEL assay

To examine the proliferative activity of cells adjacent to the surgical wounds, proliferative cell nuclear antigen (PCNA) expression were measured by immunohistochemistry. Tissues were imbedded in paraffin, 4 μm thick sections from eyes at 28 days post-operation were used. After deparaffinization, the tissues were mounted on poly-L-lysine-coated slides. The sections in a citrate buffer (0.01 mol/l, pH 6) were heated in a microwave oven for 15 min at maximum power (700 W) and then cooled at room temperature for 20 min. Tissues were blocked by 1% BSA first and then incubated with PCNA primary antibody (1:700; Millipore, Boston, MA) at room temperature for 2 h. After washing with PBS, slides were then incubated with biotinylated goat anti-polyvalent antibody for 15 min and then in streptavidin peroxidase for 15 min. The slides were developed for 12 min in diaminobenzidine.

For apoptotic cells analysis around the surgical wounds, the terminal deoxynucleotidyl transferasemediated dUTP nick end labeling (TUNEL) method was used according to the manufacturer’s specifications (In Situ Cell Death Detection Kit; POD; Roche Applied Sciences, Indianapolis, IN). Nuclei were counterstained with hematoxylin.

The percentages of PCNA and TUNEL positive cells were calculated by a computer-assisted morphometric analysis. Approximately 1,000 cells or nuclei were counted for each section, 4 sections in each rabbit were analyzed.

### Statistical analysis

SPSS 14.0 (SPSS Inc. Chicago, IL) was used for statistical analysis. Numerical variables with normal distribution are described by the mean±standard deviation (mean±SD), non-normal distribution is described by median and interquartile range, and categorical variables are described by the number of cases (n) and its percentages. Comparison of differing times of IOP observation within the same group, comparison of corneal endothelial cell count pre-op and post-op among groups with differing treatment, and comparison of corneal endothelial cell count pre-op and post-op within the same group is done with one-way ANOVA. If there are significant differences in pairwise comparisons the LSD-*t*-test used. Comparison of filtering bleb sustained time between groups with differing treatment is performed using the Kaplan–Meier analysis. Test of significance is performed by the log rank analysis. If p<0.05 bilaterally, it was considered statistically significant.

## Results

### Postoperative IOP changes

**Figure 2:**
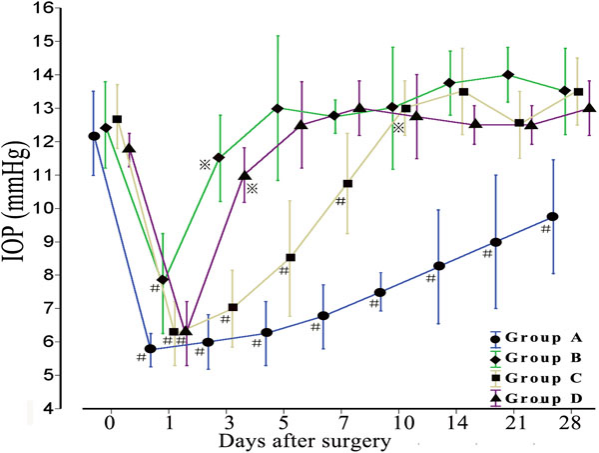
Postoperative IOP changes during a 28 days period in each group. Across all groups, IOP was at its lowest day 1 post-op, with upward trends starting day 3. Group B and Group D had sharp increases on day 3, surpassing 12 mmHg on day 5, reaching an equilibrium soon after. Group C had a slower increase, surpassing 12 mmHg on day 10, and then reaching equilibrium. Group A continually sustains a lower IOP than pre-op during the observed period. ^#^p<0.05, ^※^p>0.05 versus pre-op (n=4).

The average IOP among the four groups at day 1 post-op showed a significant decrease compared to pre-op, and had no statistic differences between groups. At day 3, Groups B and D showed a rapid increase in IOP, reaching a general equilibrium point at day 5. In Group C, IOP began to increase at day 5, reaching pre-op levels in 10 days. While at day 28, Group A still maintained lower average IOP, with 9.75±1.71 mmHg, showing significant difference when compared to pre-op levels (p<0.001; Figure 2).

### Clinical evaluations and complications

At day 1 post-op, all four groups had similar average levels of conjunctiva congestion. Group A and Group C began showing slow relief of conjunctiva congestion starting at day 3, while Group B and Group D began showing slow relief of conjunctiva congestion around day 5. One subject of Group C had severe corneal edema at day 1 post-op, however it gradually receded after 3 days, with no significant corneal edema in the remaining three groups. All four groups experienced mild anterior chamber flare at day 1, with gradual relief around day 3. In Group C and Group D, each had 1 case of hyphema at day 1, both generally resolving after 5 days. There were no observable signs of endophthalmitis, filtering bleb leakage, cataract and other complications among all four groups in 28 days observation.

### Filtering bleb and survival curve

**Figure 3:**
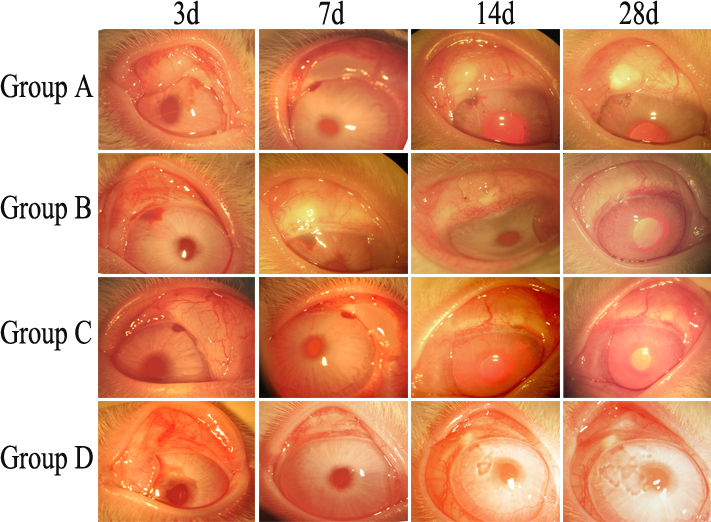
Post-op morphological changes to filtering bleb. Group B and Group D was sustained for less than 3 days, and Group C for 14 days. Group A filtering bleb sustained for greater than 28 days.

**Figure 4:**
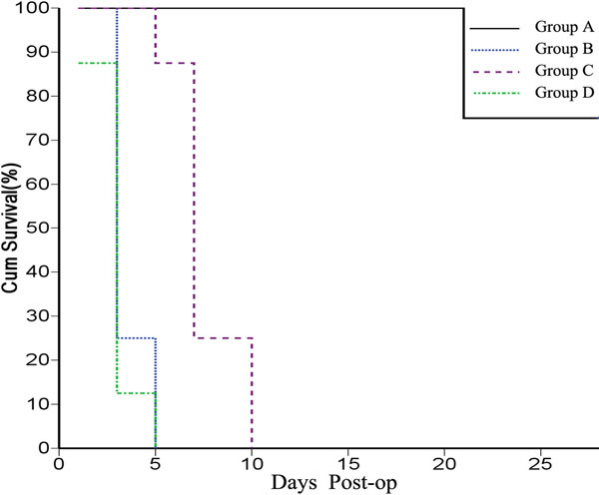
Survival curve of the filtering bleb of each group (n=8). In Group B and Group D, filtering blebs failed within 5 days post-op, with a median survival of 3 days. Group C filtering blebs failed within 10 days post-op, with a median survival of 7 days. Group A had 6 filtering blebs still sustained by the end of the observation period.

At post-op day 1, all four groups showed diffuse filtering bleb swelling with obvious congestion. In Group B and Group D, swelling had generally resolved at day 3, however congestion remained, and filtering blebs failed within 5 days post-op, with a median survival of 3 days. In Group C, swelling began to shrink and become localized at day 7, with obvious reduction in congestion, filtering blebs failed within 10 days post-op, with a median survival of 7 days. In Group A, swelling began to shrink and become localized at day 7 also, but at day 14 it maintained a stable level, and at day 28 it presents as localized bulged filtering bleb in 6 rabbits (Figure 3 and Figure 4).

Kaplan–Meier analysis shows a significant statistical difference in the survival distributions between Group A and other groups (p<0.001), and between Group C and Group B (p<0.001), but Group B and Group D did not have statistically significant differences (p=0.340).

### Changes in corneal endothelial cell count

The corneal endothelial cell count was performed at pre-op and post-op 28 days. Table 1 showed that there were no significant changes between groups (p>0.05), and demonstrated that there were no serious side effects to corneal endothelial cells from surgical treatment and Sirolimus sustained delivery film application.

**Table 1:** Corneal endothelial cell count (/mm^2^, mean±SD) changes at pre-op and post-operation at day 28.

**Group**	**n**	**pre- operation**	**post-operation**	**t value**	**p value**
A	8	2394.1±219.6	2353.9±129.6	0.621	0.545
B	8	2415.5±188.2	2384.3±136.9	0.379	0.711
C	8	2336.2±142.1	2287.2±119.2	0.747	0.467
D	8	2374.3±192.5	2279.8±114.8	1.191	0.253
F value		0.332	1.325		
P value		0.802	0.286		

### Sirolimus concentration in aqueous humor

At post-op days 3, 7, 14, and 28, the concentration of Sirolimus in aqueous humor of Group A was (10.56±0.05) ng/ml, (8.62±0.05) ng/ml, (7.74±0.05) ng/ml, and (3.77±0.05) ng/ml. Thus, we can ascertain that the effective drug concentration in the aqueous humor can be maintained for over two weeks.

### Histological examination

**Figure 5:**
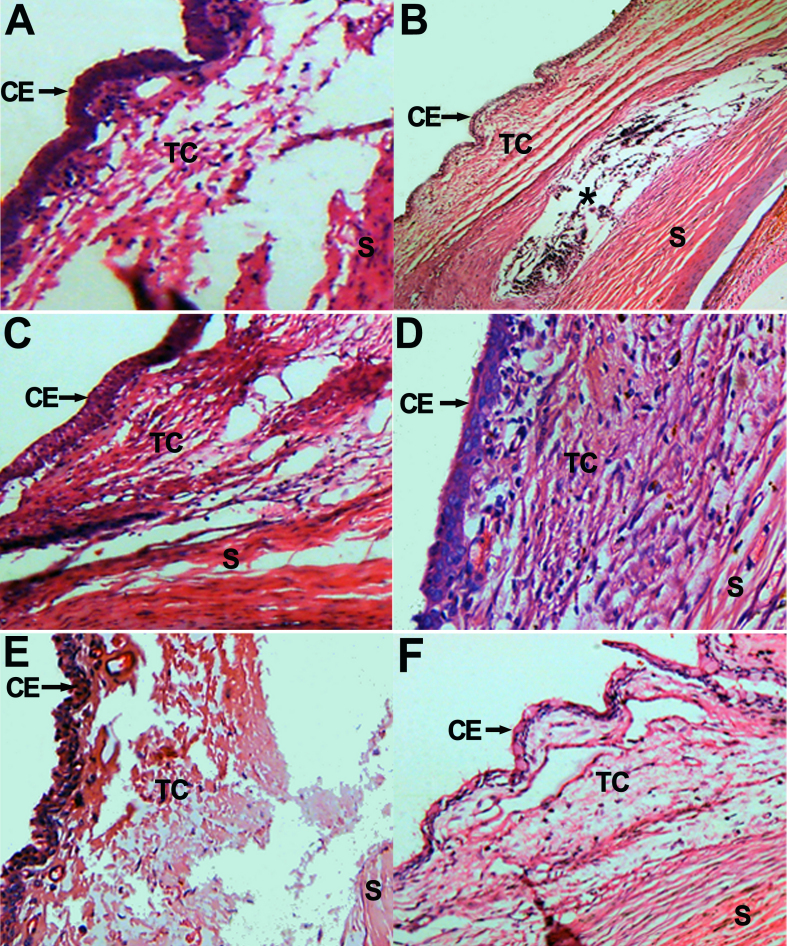
Histopathologic features of the surgical area in different groups. **A**, **E**: At day 7 and day 14 post-op in Group A, respectively, showing intact conjunctiva epithelium (CE), only mild inflammation infiltrate in subconjunctiva scleral filtration area, wide conjunctiva and scleral space (S), and small amounts of fibroblasts in Tenon’s Capsule (TC). **B**, **E**: Group B and Group D showing that conjunctival epithelium (CE) is intact, infiltration of inflammation around the subconjunctival scleral filtration area, conjunctiva flap and sclera (S) space narrowing, and dense fibroblasts hyperplasia in the Tenon’s Capsule (TC) at 7 days post-op. *represents sustained-release drug film area in **B**. **C**: Showing a moderate number of fibroblasts hyperplasia and a certain subconjunctiva space at day 7 post-op In Group C, however, **F**, shows dense fibroblasts hyperplasia in the Tenon’s Capsule (TC) and subconjunctival space almost disappear at day 14 post-op. (H-E staining, original magnification: **A** 40×; **B** 200×; **C** 200×; **D** 200×; **E** 100×; **F** 100×).

**Figure 6:**
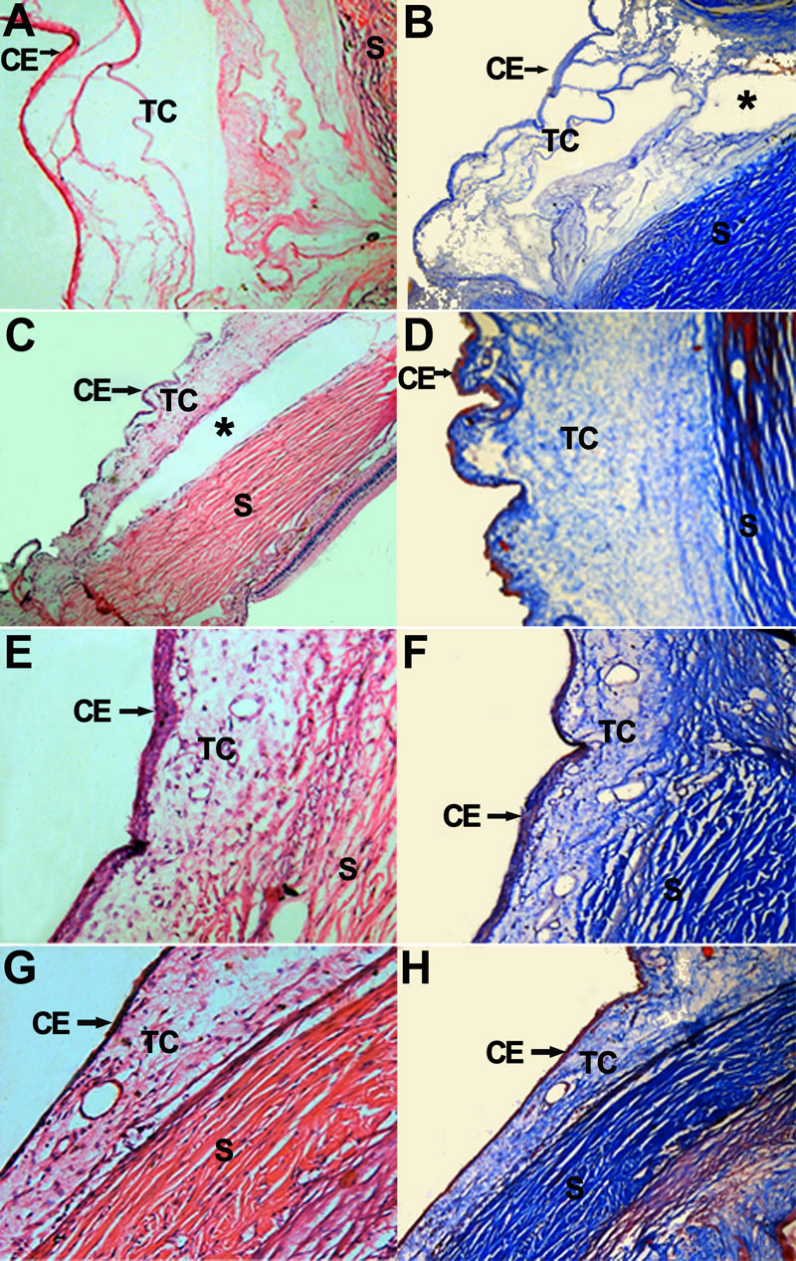
Histopathologic features of the surgical area in each group at 28 days post-op. **A**-**H** represent pathological changes in the surgical area of Group A, Group B, Group C, and Group D at 28 days post-op, respectively. Conjunctiva Epithelium (CE) in each group are in good condition, conjunctiva flap and scleral (S) space are wide in **A** and **B**, and the Tenon’s Capsule (TC) has few fibroblasts and new collagen tissue. In **C**-**H** conjunctiva flap and scleral space narrow or even disappear, and a large amount of fibroblasts hyperplasia and new collagen tissue deposition can be observed in the Tenon’s Capsule (TC). *represents sustained-release drug film area. H-E staining, original magnification: **A** 40×; **C** 40×; **E** 100×; **G** 100×. Masson trichrome staining, original magnification: **B** 40×; **D** 100×; **F** 100×; **H** 40×.

Conjunctiva epithelium was intact in each group after operation. At post-op day 7, dense fibroblast and inflammatory cell infiltration can be seen in the subconjunctival scleral filtration area in Group B and Group D (Figure 5B,D), but at day 14 and day 28, there were no infiltrate could be observed. The narrowing or disappearance of the subconjunctival space is related directly to increase of IOP. At post-op day 7, 14, and 28, the filtration area under the conjunctiva and sclera showed large amounts of fibroblasts hyperplasia and dense new collagen tissue deposition. At post-op day 7 Group C showed a certain subconjunctival space, a moderate number of fibroblasts hyperplasia and new collagen deposition, the subconjunctival space disappeared by days 14 and 28, with subconjunctival and filtration area showing large amounts of fibroblasts hyperplasia (Figure 5C,F). At post-op day 7, 14, and 28, Group A showed clear subconjunctival space and a small number of fibroblasts and new collagen tissue (Figure 5A,E and Figure 6A,B). While in other groups, subconjunctival spaces had disappeared and already formed dense scar tissue by day 28 (Figure 6C-H). Retina and ciliary body remained intact in all groups, and no effusion or detachment was observed.

### PCNA and TUNEL findings

**Figure 7:**
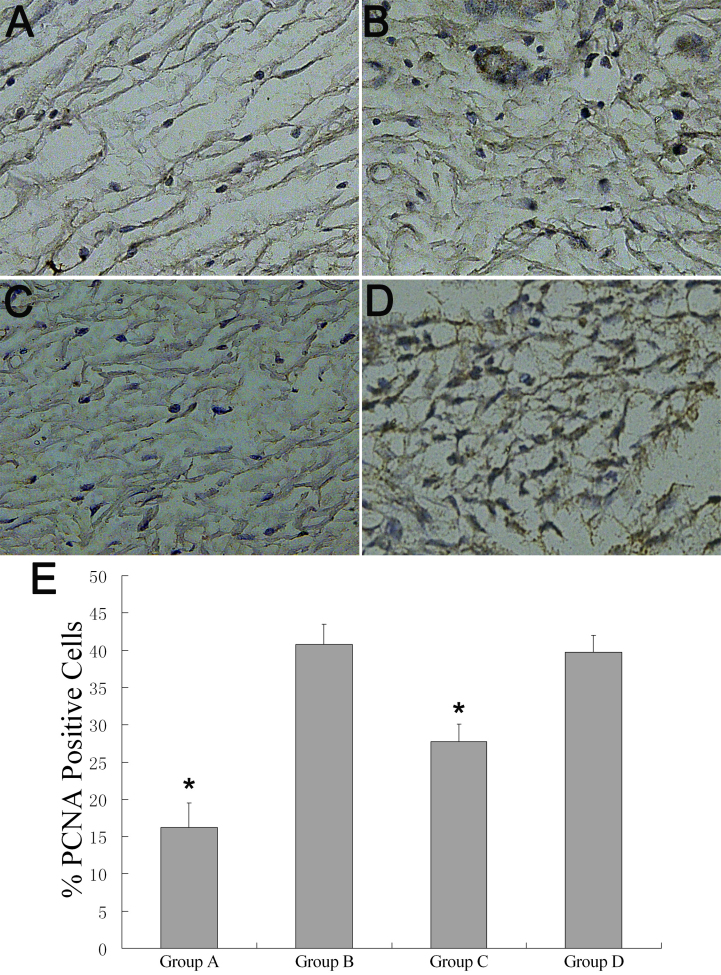
Proliferating cell nuclear antigen (PCNA) analysis in each group at 28 days post-op. **A**-**D**: Immunohistochemistry for PCNA: **A**: Group A, **B**: Group B, **C**: Group C, **D**: Group D. **E**: Quantitation of the number of PCNA positive cells in the subconjunctival space at post-op 28 days. *p<0.01 for treated (rapamycin) versus control (Group B and D; n=4). Original magnification: 200×.

PCNA-expression was lower in the Group A (16.25±3.24%) as compared to other groups (B=40.75±2.75%, C=27.75±2.36%, D=39.75±2.22%; n=4, p<0.01), and it was lower in Group C than Group B and D (p<0.01; Figure 7).

**Figure 8:**
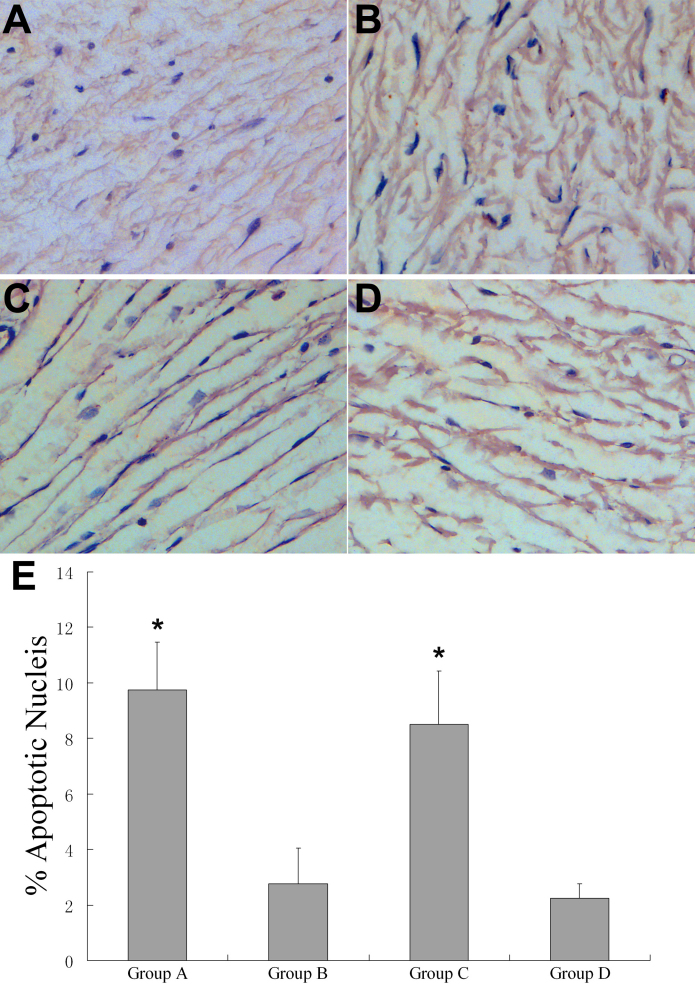
Apoptosis by TUNEL assay in each group at 28 days post-op. **A**-**D**: Immunohistochemical analysis for apoptosis by TUNEL assay. **A**: Group A, **B**: Group B, **C**: Group C, **D**: Group D. **E**: Quantitation of the number of apoptotic fibroblasts in the subconjunctival space at post-op 28 days. *p<0.01 for treated (rapamycin) versus control (Group B and D; n=4). Original magnification: 200×.

TUNEL assay showed a significant increase in the number of apoptotic fibroblasts around the surgical area in Group A and Group C (9.75±1.71% and 8.50±1.92%) as compared to the other groups (B=2.75±1.29%, D=2.25±0.50%; n=4, p<0.01). There was no significant difference in Group A and Group C (p>0.05; Figure 8).

The results indicated that rapamycin could inhibit the proliferation of fibroblasts and induce its apoptosis around the surgical region in rabbits.

## Discussion

Filtration surgery is one of the most effective treatment methods for glaucoma patient, but often failed due to excessive scarring. Currently, first line perioperative anti-proliferative drugs, include MMC, 5-FU, etc, have improved the success rate of the surgery. But the toxicity of existing drugs and the drug delivery system are still questionable. Usually, the surgeons placed a medication-soaked sponge pad above and/or below the scleral flap for a certain time, and then washed out with PBS. Because these drugs have an immense inherent cytotoxicity, and the delivery system is not sustained, there is a great disparity in drug concentration and uncontrolled complications [17-21]. In addition, it had been well established that the active proliferation of fibrosis during incision healing of filtration surgery can last up to two weeks after surgery [22]. Apparently, the time of MMC and 5-FU immersion is deficient, and the efficacy of the anti-metabolism treatment is transient, and is not ideal for long-term anti-proliferation treatment. Therefore, finding an effective, long lasting treatment with low toxicity is an important aspect of glaucoma research.

Sustained drug delivery system (DDS) is a usefully way to get stable, effective, and long-term drug treatment effects. Currently, DDS including eye implants, liposomes, biodegradable polymers, and electric pulse directed drug delivery systems, etc [23-29]. Polyactic acid (PLA) and its derivatives has been a significant progress in the field of medical study. PLA does not accumulate in the body, has no immunogenicity or antigenicity, and does not have side effects on local tissue or the body at large [30-35]. The copolymer of PLA and polyglycolic acid (PGA), polymer polylactioglycolic acid (PLGA) has good biocompatibility [36]. By changing the percentage of the content in PLGA (PLA or PGA), the time of degradation and rate of drug release can then be adjusted. The degradation of the PLGA surface forms porous surfaces that constantly deliver its internal encapsulation of drugs, and thus can be used as an implantable carrier [37-39]. In the present study, the PLGA film which synthesis by PLGA50:50poly(DL-lactide-co-glycolide) inherent viscosity 0.17 dl/g and PLGA50:50poly(DL-lactide-co-glycolide) inherent viscosity 0.55–0.75 dl/g, was starting to degenerated by 2 weeks, and then absorbed and degenerated day by day until 2 months. This degeneration time make sure the Sirolimus concentration maintain constant until the bleb formation.

Sirolimus, a new, potent, low toxicity macrolide, has been demonstrated that primarily inhibit the mammalian target of rapamycin (mTOR), thus interfering with the phosphoinositide 3-kinase (PI3K)-Akt-mTOR axis, that is a key signal pathway to modulate cellular differentiation, viability and growth [7,40]. Some reports also stated that Sirolimus is a potential inhibitor that blocked the proliferation of several kinds of cells at the Gl restriction point [7-10]. Salas-Prato et al. [11] illustrated that Sirolimus potently inhibits platelet-derived growth factor (PDEF) and basic fibroblast growth factor (b-FGF) induced fibroblast proliferation on human Tenon’s fibroblast proliferation after glaucoma filtration surgery, and was not cytotoxic at any of the concentrations tested. These findings give us new horizon on the anti-proliferation study on filtration surgery.

Here, we used Sirolimus applied via a PLGA drug sustained delivery film, not only solved the Sirolimus related problems, such as limited solubility, easy precipitation, and instability at room temperature, but also get a stable release of the medication, achieving an effective local concentration. In our study, we demonstrated that the film itself had good in vivo biocompatibility and carries no significant ocular toxicity (Group B). After implantation under conjunctiva, there was no significant inflammation, tissue necrosis, or other adverse reactions, and no effect on corneal endothelium or retina. In Siromilus treated group (Group A and C), they maintained a low IOP for a longer time comparing to no treatment group (Group D), and post-op inflammatory infiltration is mild, and no toxic effects were observed on the tissue tested. Furthermore, the sustained Siromilus delivery group (Group A) analysis revealed that there was a sustained drug release concentration preserved over 2 weeks, and maintained low IOP and filtering bleb for a much longer period of time, and had milder post-op inflammation. In addition, the pathological results showed that the subconjunctival space of Group A was wide at days 7, 14, and 28 post-op, with small amounts of fibroblasts hyperplasia and new collagen tissue deposition, and inflammatory infiltrate around the surgical area was lighter. According to the histological examination of our study and other published articles, fibroblasts are most active in the first 2 weeks following the glaucoma filtration surgery [22]. PLGA carrier can deliver the Sirolimus to the surgery site for a period of time in excess of 2 weeks [40], therefore it meets the requirements of anti-proliferation treatment strategy. Although Sirolimus delivery system showed no significant inflammation response or toxicity in this study, the effect is limited in terms of time.

In addition to the histology studies above, we try to understand the mechanisms of Sirolimus during anti-scaring formation course. It had been proved that PCNA (proliferating cell nuclear antigen) first expressed in mid-G1, and plays important role of the principle of fibroblast cell proliferation [41]. In our study, PCNA-expression was decreased in Sirolimus treated groups, indicated that the proliferative activity were impeded maybe through blocking cell cycle in the G_1_ Phase [42]. Also, we demonstrated that Sirolimus can induce fibroblast apoptosis (TUNEL assay) in filtration site for the first time in rabbit model. Consequently, Sirolimus susteained dilivery system may have great potential to significantly improve the success rate of glaucoma filtration surgery.

In summary, Silolimus sustained delivery film was proven to be able to safely and effectively inhibit inflammation and fibroblast proliferation surgery in rabbit eyes during glaucoma filtration surgery, preventing filtering bleb scarring and increasing the success rate of filtration surgery. This makes Silolimus a very promising new drug in the field of glaucoma research.
